# Impact of Percutaneous Endoscopic Gastrostomy (PEG) on the Evolution of Disease in Patients with Amyotrophic Lateral Sclerosis (ALS)

**DOI:** 10.3390/nu13082765

**Published:** 2021-08-12

**Authors:** Juan J. López-Gómez, María D. Ballesteros-Pomar, Beatriz Torres-Torres, Begoña Pintor-De la Maza, María A. Penacho-Lázaro, José M. Palacio-Mures, Cristina Abreu-Padín, Irene Sanz-Gallego, Daniel A. De Luis-Román

**Affiliations:** 1 Servicio de Endocrinología y Nutrición, Hospital Clínico Universitario de Valladolid, 47003 Valladolid, Spain; beatriztorrestorres@hotmail.com (B.T.-T.); dadluis@yahoo.es (D.A.D.L.-R.); 2 Centro de Investigación Endocrinología y Nutrición de Valladolid (CIENC), Universidad de Valladolid, 47003 Valladolid, Spain; mdballesteros@telefonica.net; 3 Sección de Endocrinología y Nutrición, Complejo Asistencial Universitario de León, 24008 León, Spain; bpintor.asitec@saludcastillayleon.es; 4 Servicio de Endocrinología y Nutrición, Hospital de El Bierzo, 24404 Ponferrada, Spain; penacholazaro@gmail.com; 5 Servicio de Endocrinología y Nutrición, Hospital Universitario Rio Hortega, 47012 Valladolid, Spain; jmpalaciomures@hotmail.com; 6 Servicio de Endocrinología y Nutrición, Complejo Asistencial de Segovia, 40002 Segovia, Spain; cabreupadin@gmail.com; 7 Servicio de Neurología, Complejo Asistencial de Ávila, 05071 Ávila, Spain; irenesanzgallego@gmail.com

**Keywords:** amyotrophic lateral sclerosis, percutaneous endoscopic gastrostomy, nutritional status, survival

## Abstract

Dysphagia is a highly prevalent symptom in Amyotrophic Lateral Sclerosis (ALS), and the implantation of a percutaneous endoscopic gastrostomy (PEG) is a very frequent event. The aim of this study was to evaluate the influence of PEG implantation on survival and complications in ALS. An interhospital registry of patients with ALS of six hospitals in the Castilla-León region (Spain) was created between January 2015 and December 2017. The data were compared for those in whom a PEG was implanted and those who it was not. A total of 93 patients were analyzed. The mean age of the patients was 64.63 (17.67) years. A total of 38 patients (38.8%) had a PEG implantation. An improvement in the anthropometric parameters was observed among patients who had a PEG from the beginning of nutritional follow-up compared to those who did not, both in BMI (kg/m^2^) (PEG: 0 months, 22.06; 6 months, 23.04; *p* < 0.01; NoPEG: 0 months, 24.59–23.87; *p* > 0.05). Among the deceased patients, 38 (40.4%) those who had an implanted PEG (20 patients (52.6%) had a longer survival time (PEG: 23 (15–35.5) months; NoPEG 11 (4.75–18.5) months; *p* = 0.01). A PEG showed a survival benefit among ALS patients. Early implantation of a PEG produced a reduction in admissions associated with complications derived from it.

## 1. Introduction

The incidence of amyotrophic lateral sclerosis (ALS) in Europe and North America is between 1.5 and 2.5 people per 100,000 inhabitants/year, while the prevalence ranges between 2.7 and 7.4 cases per 100,000 inhabitants [1,2]. The incidence is slightly higher in men, 1.3–1.5:1; and increases with age, with a peak around 70 years. Survival of this disease from diagnosis is 20% at five years after diagnosis [3].

Currently there is no curative treatment and the prognosis of the disease is poor [3]. However, there are effective measures to increase the quality of life of patients and prolong their survival, including ensuring and maintaining a correct nutritional status from the initial stages of the disease to prevent the appearance of medical complications related to malnutrition and especially with the protein deficit [4]. All this is carried out by a multidisciplinary team that includes professionals from different fields of health knowledge.

One of the causes that favors weight loss in patients with ALS, and, therefore, compromises their survival, is the development of dysphagia. Dysphagia appears in approximately 60% of patients with ALS [5], and in many cases, it is the initial symptom and constitutes one of the most serious complications in these patients. Dietary advice in dysphagia is given to facilitate swallowing, optimize nutritional intake and decrease the risk of aspiration. Initially, we carry out non-invasive measures such as swallowing rehabilitation or the adaptation of the consistency of food through thickeners and porridges [6]. In cases in which the ability to eat is clearly compromised, we consider more invasive methods such as the placement of a gastrostomy tube.

The most widely used technique is percutaneous endoscopic gastrostomy (PEG), which is a surgical procedure performed under endoscopic control. The current guidelines recommend the placement of a percutaneous endoscopic gastrostomy (PEG) in ALS when the patient’s forced vital capacity is still greater than 50% [5], since a lower percentage would indicate a greater deterioration in lung function and, therefore, an increased risk of complications during the procedure. Generally, the placement and use of the PEG tube is well tolerated by the patient and allows them to meet their nutritional needs. However, it could have some complications that must be taken into account [7].

The early implantation of the gastrostomy has not clearly demonstrated a benefit in the survival of patients, although it can improve the quality of life of these and prevent the development of complications of the disease [4]. However, when the oral route is possible, both the patient and their relatives are usually reluctant to implantation. Therefore, most of the clinical guidelines recommend the decision of early PEG implantation in a consensual manner with the patient and family, with an adequate assessment of its advantages and disadvantages [8,9].

ALS is a disease with a highly variable course that depends on individual characteristics. The nutritional status in this disease has been shown as the basis for survival in these patients [10]. It is, therefore, important to develop evidence on methods that can preserve this nutritional situation, such as a gastrostomy. In addition, it is essential to know if gastrostomy implantation can influence survival in these patients and which patients can benefit from it.

The aims of this study were to describe the characteristics of patients with ALS who underwent PEG implantation and to assess the complications and survival in patients with and without an SGA.

## 2. Materials and Methods

### 2.1. Design

A prospective observational cohort study (patients with a PEG (PEG) and patients without a PEG (NoPEG)) was designed to evaluate the nutritional status at the beginning of follow-up by the Nutrition Unit and the effect of the nutritional status on survival.

Those patients included in the study were referred to the Clinical Nutrition Unit with a diagnosis of motor neuron disease in six hospitals in the Castilla-León region (Spain) between January 2015 and December 2017.

A protocol based on clinical guidelines has been implanted in hospitals of the Castilla-León region (Spain) since September 2015 to improve the multidisciplinary attention of these patients. The patients begin follow up in Clinical Nutrition Units at diagnosis of ALS, regardless of the nutritional status or nutrition-related symptoms. The specialist in endocrinology and nutrition carried out the nutritional assessment and the patient-based treatment was made [11].

### 2.2. Procedures

After signing the informed consent and including the patient in the study, the patients were included in a web platform created for this purpose, on the website of the Center for Research in Endocrinology and Nutrition (www.ienva.org (accessed on 2 June 2021)), in compliance with the Law of Data protection LOPD15/1999. This study was carried out in accordance with the Declaration of Helsinki, and all procedures were approved by the Research Committee of the Hospital Clínico Universitario de Valladolid with the code PI 17-543. The variables were recorded at the beginning of the nutritional support and every six months during the follow-up of the patient in the Clinical Nutrition Unit.

With the data obtained, a descriptive statistical analysis of prevalence and the nutritional status of the patients was performed. Subsequently, a univariate and multivariate inferential statistical analysis was carried out to evaluate the evolution of the disease, as well as the effect of nutritional support on the proposed outcome variables.

### 2.3. Variables

#### 2.3.1. Characteristics of the Disease

Sex, date of birth and type of amyotrophic lateral sclerosis (ALS), characterized by the onset form, were collected. In this way, patients were classified as spinal-onset ALS and bulbar-onset ALS. The diagnosis of ALS and other motor neuron disease was made in terms of Airlie House and revised El Escorial Criteria [12].

To characterize the evolution of the disease, the date of the onset of symptoms, the date of diagnosis of the disease made in the Neurology Service and the date on which it was assessed for the first time in the Endocrinology and Nutrition Service, were collected. In addition, income associated with the disease was assessed. The death rate and the date on which they occurred were also analyzed.

To analyze the evolution of dysphagia treatment, the dysphagia onset date, the type of gastrostomy implanted and the date it was implanted were assessed. Likewise, the type of nutritional support treatment prescribed, the complications associated with nutritional treatment (diarrhea, constipation, aspiration pneumonia and admissions related to PEG) were also assessed.

#### 2.3.2. Implantation of PEG

The intervention evaluated was percutaneous endoscopic gastrostomy (PEG) implantation. This technique is performed through a minor surgical intervention in which a gastrostomy tube was inserted percutaneously through endoscopy by the gastroenterology service. The patient was admitted one day before the intervention and nutritional support was started through the gastrostomy tube 4 h after implantation.

A comparison of the different variables was made between those patients in whom PEG was implanted and those in whom it was not implanted. Among those patients in whom PEG was performed, the variables were compared in those patients in whom it was implanted in the 10 months after diagnosis of the disease, and in those in whom it was implanted later. The 10-month limit was chosen given that it was the median follow-up until PEG implantation. The choice of this value was made to assess the differences as a function of time to implantation. This cut-off point (10 months) was due to sample dispersion criteria. 

#### 2.3.3. Anthropometry

The anthropometric evaluation of the subjects was carried out by determining the weight, height and body mass index.

Weight was measured with an accuracy of ±0.1 kg with a scale to the nearest 0.1 kg (SECA, Birmingham, UK). Height was measured with the patient standing with a height rod (SECA, Birmingham, UK). The body mass index (BMI) was calculated using the formula: weight (kg)/height × height (m^2^).

If the patient came in a wheelchair and could not move, the weight was measured indirectly (weight of the patient in the chair—weight of the wheelchair). Height in these cases was determined by ulnar distance.

The percentage of weight loss (%WL) was used to assess the relative difference in weight.

#### 2.3.4. Nutritional Assessment

The nutritional risk of the patients was measured using the subjective global assessment (SGA). This is a simple test for the diagnosis and categorization of malnutrition that integrates variables from the history, physical and biochemical examination. This test is subdivided into the following three groups: A, good nutritional status; B, moderate malnutrition; C, severe malnutrition [13].

### 2.4. Data Analysis

The data were stored in a database of the statistical package SPSS 15.0 (SPSS Inc. Chicago, IL, USA) officially licensed by the University of Valladolid. A normality analysis of the continuous variables was performed with the Kolmogorov–Smirnov test.

Continuous variables were expressed as mean (standard deviation) and non-continuous variables as median (p25–p75). Parametric variables were analyzed with the unpaired and paired Student’s *t*-test, and non-parametric variables with the Friedman, Wilcoxon, K Kruskal and U-Mann–Whitney tests.

The qualitative variables were expressed as percentages (%) and analyzed with the chi-square test (with Fisher and Yates corrections when necessary).

A survival analysis was performed based on PEG implantation by means of a log-rank test and the Kaplan–Meier curves.

A multivariate analysis with a Cox model was made to evaluate the relationship of malnutrition with PEG or no PEG implantation.

## 3. Results

A total of 93 patients with a diagnosis of amyotrophic lateral sclerosis (ALS) were analyzed. Among the patients with ALS, 49 patients (52.7%) had symptoms of spinal onset, and in 44 patients (47.3%) they had symptoms of bulbar onset. The patients with motor neuron disease without ALS, according to the Airlie House criteria, were excluded from analysis, as these patients had a different evolution of the disease.

The contribution of patients from the different hospitals in the Castilla y Léon region was as follows: Complejo Asistencial de Ávila, 1 patient (1%); Complejo Asistencial Universitario de León, 30 patients (30.6%); Hospital de El Bierzo, 14 patients (14.3%); Complejo Asistencial de Segovia, 1 patient (1%); Hospital Clínico Universitario de Valladolid, 42 patients (42.9%) and Hospital Universitario Rio Hortega de Valladolid, 10 patients (10.2%).

**Figure 1:**
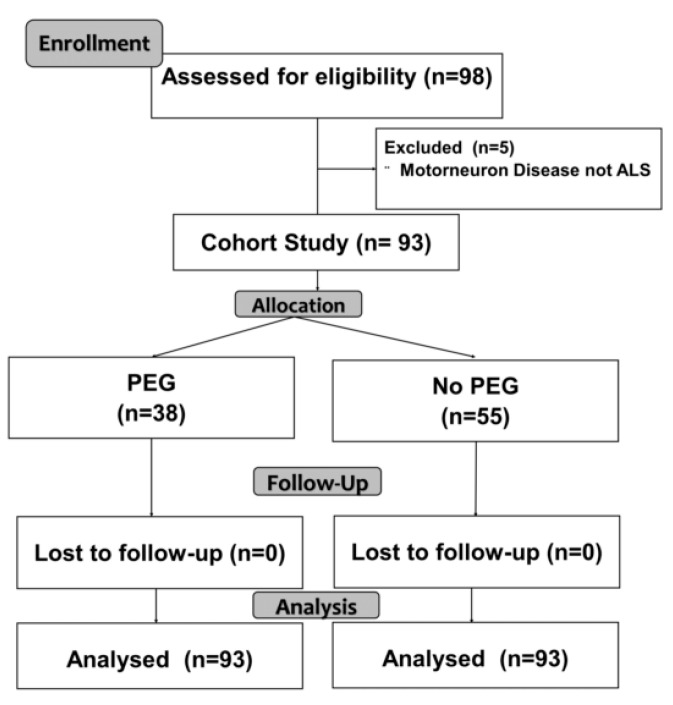
Flow Chart.

At the beginning of nutritional support, 82 patients (83.6%) kept the oral route, and 16 patients (33.7%) were gastrostomy carriers (33.7%). Of the total number of patients analyzed, a PEG was implanted in 38 patients (38.8%) (Figure 1, flow chart).

The mean age of the patients was 64.63 (17.68) years; 57 (58.2%) were men and 41 (41.8%) were women. The median follow-up at the nutrition clinics was 12 (6–20) months. Of the total number of patients, 73 patients (78%) developed dysphagia during the study. The median time from diagnosis to development of dysphagia was 0 (0−54) months. The median time from diagnosis to PEG implantation was 10 (3–17.25) months. The mean time from the first symptoms to PEG was 21.5 (12.75–32.25) months. The mean time from the onset of symptoms to the first visit to the Nutrition Unit was 13 (8–28.5) months. A total of 18 (19.4%) patients needed non-invasive ventilation; in those patients with a PEG, four (4.3%) were in treatment with non-invasive ventilation before the implantation of gastrostomy. 

A total of 73 (78.5%) patients developed dysphagia; 32 (91.4%) patients had dysphagia prior to PEG implantation.

The differences between the patients with and without PEG are shown in Table 1.

**Figure 2:**
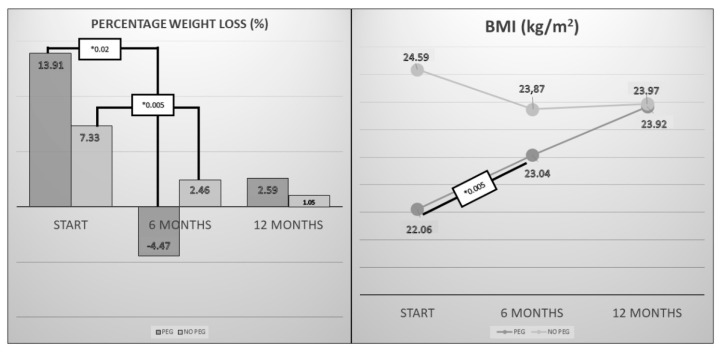
Changes in the percentage of weight loss and body mass index (BMI) since the start of nutritional support in those patients with Percutaneous Endoscopic Gastrostomy (PEG) from the start and those who did not. * *p* < 0.05.

We analyzed the modification of the anthropometry in relation to the presence or absence of feeding gastrostomy at the beginning of the nutritional support follow-up. It was observed that those patients with gastrostomy at the beginning of nutritional follow-up (*n* = 16) had a lower BMI (initial PEG, 21.83 (6.27) kg/m^2^; No initial PEG, 24.40 (3.44) kg/m^2^; *p*-value < 0.01) and greater weight loss since diagnosis (baseline PEG, 16.32% (11.08); No baseline PEG, 8.62% (8.86); *p*-value 0.03). However, it was observed that the change in weight and BMI was more favorable in these patients (Figure 2).

A PEG was implanted early (less than 10 months after diagnosis) in 19 patients (50%); while it was implanted late (more than 10 months after diagnosis) in 19 patients (50%) (Table 2). 

In patients in whom a PEG was implanted late (more than 10 months after diagnosis), 27.8% had some type of gastrostomy-related complication, while in those implanted earlier (less than 10 months after diagnosis) complications did not present (*p* < 0.01). In the case of respiratory failure (due to the evolution of the disease or bronchoaspiration), no differences were observed (*p* = 0.92) between the patients with early implantation (26.30%) versus late implantation (27.30%).

**Figure 3:**
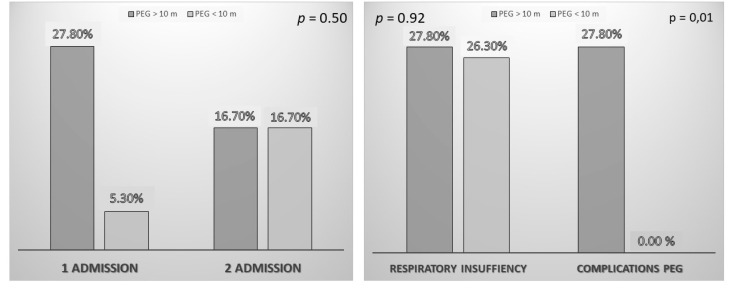
Differences in the percentage of admissions and the cause of admissions depending on the implantation of PEG (percutaneous endoscopic gastrostomy) before (PEG < 10 months) or after (PEG > 10 months) 10 months from the diagnosis of ALS (amyotrophic lateral sclerosis).

When evaluating the admissions of the patients and the cause of these, a higher rate of admissions in relation to complications of PEG was observed in those patients in whom it was implanted later. No other significant differences were observed, although there was a trend toward an increase in admissions in those who had a PEG implanted later (Figure 3).

Of the total number of patients, 38 (37.8%) died during the follow-up period, the median time between diagnosis and death was 16 (8.75–27.5) months. The disease duration was 26 (16–42) months (Patients who died: 28 (17–41) months and patients who did not die: 25 (15–43.5) months).

**Figure 4:**
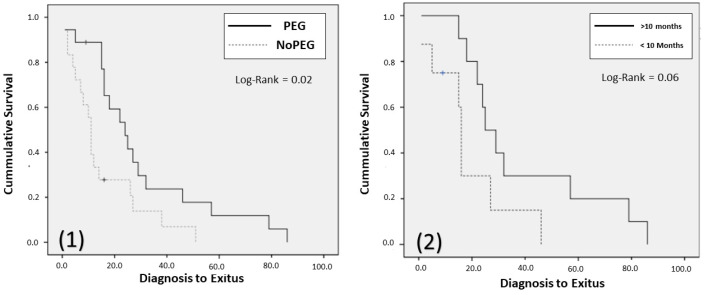
Kaplan–Meier curves: (**1**) Comparing survival in patients deceased in function of PEG implantation or not. PEG: Percutaneous Endoscopic Gastrostomy. (**2**) comparing survival in patients deceased in function of PEG early implanted (<10 months) or not (>10 months). PEG: percutaneous endoscopic gastrostomy.

Among those patients whose outcome was death (*n* = 38; PEG = 20), a survival analysis was performed, comparing those who had a gastrostomy implant versus those who did not. A significant survival advantage (PEG, 23 (15–35.5) months; NoPEG 11 (4.75–18.5) months; *p* = 0.01) was observed among those in whom a gastrostomy was implanted (Figure 4).

When evaluating survival based on early PEG implantation (less than 10 months after diagnosis), a longer survival was observed, although not significant in those patients in whom a PEG was implanted later (PEG (>10 months), 27 (21–62) months; PEG (<10 months), 15.5 (6–24.25) months; *p* = 0.06) (Figure 4).

When performing the multivariate analysis, it was observed that the probability of survival above 12 months from the start of nutrition follow-up was independently related to gastrostomy implantation (OR, 6.8; 95% CI (2.05–22.68)) adjusting for the type of onset of ALS, age and nutritional status.

When performing the univariate and multivariate analysis, no relationship was observed in the probability of survival above 12 months from the start of nutrition follow-up and the early implantation of an SGA (<10 months) when adjusting for age, type of ALS and nutritional state at the beginning of nutritional support (Table 3).

## 4. Discussion

In this multicenter study carried out in the Castilla-León region in Spain on patients with amyotrophic lateral sclerosis, the implantation of a PEG showed an improvement in nutritional parameters in those patients in whom nutritional follow-up was started at the beginning. On the other hand, among deceased patients, those in whom PEG is implanted have a survival benefit. The early implantation of this feeding method did not increase the complication rate, although it did not demonstrate a survival benefit.

The most widely used feeding route in patients with ALS is the gastrostomy tube, considering the difficulties of combining nasoenteric tubes with other treatments (such as non-invasive ventilation). The indication for gastrostomy implantation is given by dysphagia (inability to hydrate, or to consume medication) or weight loss (more than 5% in 3 months; or more than 10% in 6 months) [4]. In our study, the development of dysphagia was observed in most of the patients (78%), and a PEG was implanted in 38.8% of the patients. This situation may be due to differences in the progression of the disease. Thus, in studies such as that of Johnson et al., it is noted that the maintenance or not of the pleasure of eating is associated with a higher or lower rate of gastrostomy implantation [14]. In the same way, the performance of specialized nutritional follow-up may be related to a longer delay in the implantation of the PEG when adapting the oral diet that is more adequate and that maintains the nutritional parameters [15].

The nutritional support and the evaluation of nutritional requirements started in the first visit. This study was conducted based on a multidisciplinary protocol. The patients were referred at the diagnosis of ALS, despite the nutritional symptoms. This protocol allows us to initiate nutritional support at an early phase and patients can have a better nutritional status [16].

When evaluating the improvement in the nutritional parameters, the body mass index and the percentage of weight loss were used. In the first six months of nutritional follow-up, it was observed that patients who had an SGA from the beginning had an improvement in BMI and an improvement in weight loss compared to those who did not have an SGA. The start of nutritional follow-up was used to assess this point as it was the most comparable moment within a disease with a highly variable progression. In general, the implantation of a PEG is associated with an improvement in the nutritional parameters, Körner et al. demonstrated that 76.9% of the patients with a PEG maintained or gained weight, in a similar way to what was shown in our study [17].

In general, the indication for PEG is performed in most cases when there is a weight loss greater than 10% or significant dysphagia [18]. The delay from diagnosis to PEG implantation varies depending on the center but averages at about 12 months [19]. In our series, the median of implantation from diagnosis is similar (about 10 months), and this varies depending on the symptoms and the wishes of the patients. In this sample, most of the patients in which a PEG was implanted have dysphagia. There were no differences in the presence of dysphagia between the groups. The severity of dysphagia can explain the indication of PEG implantation.

When evaluating the patients who died during the study, the presence or absence of percutaneous endoscopic gastrostomy and its possible association with survival were assessed. A significant survival advantage was observed in gastrostomy patients at any time of evolution of disease. Other studies, such as that by Pena et al., showed an increased median survival up to 7.5 months with no differences in the mode of initiation [20]. Given that survival can be influenced by different factors such as the patient’s age, the type of ALS (bulbar onset is associated with lower survival [21]) or nutritional status (a worse nutritional status at diagnosis is associated with worse survival [10]), a multivariate analysis was carried out in which it was observed that PEG implantation is related to a survival greater than 12 months. This increase in survival was also observed in a study by Czell et al. with a mean survival of 12 months after PEG implantation [22].

An important consideration is the appropriate time for gastrostomy implantation. There is no clear evidence on the survival benefit of early implantation. However, it has been observed that a better nutritional situation with less weight loss since the diagnosis before the implantation of percutaneous endoscopic gastrostomy (PEG), a commonly used technique, is associated with an increase in survival. Thus, in the ProGas study, it was observed that those patients who had lost less than 10% of their weight before gastrostomy implantation had a longer survival [23]. In the study carried out, no improvement in survival was observed in those patients in whom a PEG was implanted before 10 months from diagnosis. An advantage was observed in those in whom the PEG was implanted later, probably related to less progressive diseases. However, when analyzing only deceased patients, the sample size is small, and the variability of the sample does not allow clear conclusions to be drawn. Probably, the differences between the onset of ALS and progression of the disease can influence the results. 

The different clinical guidelines recommend the approach to the patient and the family of the early implantation of the gastrostomy, especially if there are data of malnutrition or the inability to eat and considering the possible benefits on the quality of life and patient care [7,10]. In the case of urgent PEG implantation, a higher rate of complications and a higher cost of associated admissions were observed [24]. In our study, a higher rate of first admission was observed after later implantations and similar data in repeated admissions. These data are not significant but may be related to a higher rate of complications in more advanced diseases.

On the other hand, it would be advisable to implant the gastrostomy with adequate lung capacity to reduce the complexity of the technique [25] and prevent the patient from presenting previous carbon retention that can negatively influence the prognosis [26]. Although, it has not been clearly observed that a lower lung functional capacity at the time of gastrostomy implantation could negatively influence survival, provided it is performed by an experienced team [22,27,28]. In our sample, there were no differences in the rate of respiratory complications after SGA implantation in both early and late implantation.

A much-debated aspect in the use of gastrostomies in ALS is the type of technique to use. The most used are percutaneous endoscopic gastrostomy (PEG), radiologically inserted gastrostomy (RIG) and surgical gastrostomy. The technique of choice is PEG, although if there is a deterioration in forced vital capacity, the use of a simpler technique such as GIR is recommended, although everything will depend on the experience of the professional team that implants it [8]. In our study, the technique used was PEG, given the greater experience in this technique in our centers. When comparing the two most frequently performed gastrostomy techniques (PEG and RIG) in different studies, no significant differences were observed between both techniques, neither in survival nor in complication rate. The meta-analysis by Yang et al. (2017) did not show differences in peri-intervention complications [29].

Complications of enteral nutrition through gastrostomy have been studied in multiple retrospective studies. These complications are predominantly gastrointestinal in relation to intolerance to the formula (diarrhea, constipation, etc.), followed by respiratory complications and, to a lesser extent, mechanical and metabolic [23,30,31]. In our study, we only evaluated mechanical complications related to PEG, observing a striking increase in those patients in whom a PEG was implanted later.

The strength of this study lies in complement the actual evidence of the use of PEG in patients with ALS. This study presents the protocol-based management of a neurodegenerative disease in a public health system. This management may have many implications in nutritional status. 

The main limitation of this study is the variability in the clinical picture and in the progression of the disease to make an adequate comparison and extrapolation of survival. On the other hand, conducting a multicenter study in such a complex disease produces different protocols for action and referral of patients to the nutrition consultation. It could not be assured how many patients refused the implantation of a PEG; therefore, the representativity of the sample could be compromised. Despite this point, the follow-up and treatment in the different nutrition units was carried out in a homogeneous way.

## 5. Conclusions

Patients with amyotrophic lateral sclerosis (ALS) treated in the Castilla-León Health System, in whom a percutaneous endoscopic gastrostomy (PEG) was implanted, present a better evolution in anthropometric parameters at the beginning of follow-up. PEG implantation shows a survival benefit among ALS patients in the Castilla-León region in Spain. The early implantation of a PEG protocol based was associated with a reduction in admissions associated with complications of this technique, although this early implantation did not show an improvement in patient survival. 

## Figures and Tables

**Table 1:** Differences in variables between those who had percutaneous endoscopic gastrostomy (PEG) implanted and those who did not. M, male; F, female; ALS, amyotrophic lateral sclerosis; BMI, body mass index; SGA, subjective global assessment (A: Good Nutritional Status; B: Risk of Malnutrition; C: Severe Malnutrition); L, Liquid; S, Solid; M, Mixed.

	PEG	No PEG	*p*-Value
Gender (M/F)	52.6%/47.4%	61.7%/38.3%	0.4
Age (years)	62.68 (13.33)	65.23 (8.29)	0.27
ALS Onset (Bulbar/Spinal)	57.1%/42.9%	41.4%/58.6%	0.14
% Weight Loss	10.31 (2.62–18.83)	6.93 (2.44–16.33)	0.24
BMI (kg/m2)	22.98 (21.06–25.44)	24.88 (22.48–26.05)	0.13
SGA (A/B/C)	22.9%/40%/37.1%	32.8%/50%/17.2%	0.09
Mean Time of follow-up (months)	15 (8–23)	11 (6–19.25)	0.22
% patients with non-invasive ventilation	31.4%	12.1%	0.02
% patients with dysphagia	88.6%	72.4%	0.09
% type of dysphagia (L,S,M)	54.3%/2.9%/42.9%	51.7%/5.2%/13.8%	<0.01
Time Diagnostic to dysphagia (months)	0 (−5.5–8)	−1.5 (−5.75–0.25)	0.28
Time first symptoms to Nutrition Unit (months)	14 (7–36)	12.5 (8–24.5)	0.41

**Table 2:** Differences between Percutaneous Endoscopic Gastrostomy (PEG) before (PEG < 10 months) and after 10 months (PEG > 10 months) since diagnosis of ALS. M, male; F, female; ALS, amyotrophic lateral sclerosis; BMI, body mass index; SGA, subjective global assessment.

	PEG < 10 Months	PEG > 10 Months	*p*-Value
Gender (M/F)	50%/50%	50%/50%	1.00
Age (years)	64.25 (13.23)	61.06 (14.13)	0.50
ALS Onset (Bulbar/Spinal)	87.5%/12.5%	33.3%/66.7%	0.01
% Weight Loss	10.31 (5.18–17.96)	10.53 (4.40–22.51)	0.80
BMI (kg/m^2^)	23.38 (20.33–25.57)	23.09 (21.18–25.97)	0.95
SGA (A/B/C)	18.8%/43.8%/37.5%	22.2%/38.9%/38.9%	0.95
Time Diagnostic to dysphagia (months)	−3.5 (5.45)	10.18 (20.44)	0.02

CI, confidence interval; ALS, amyotrophic lateral sclerosis; PEG, percutaneous endoscopic gastrostomy; SGA, subjective global assessment.

**Table 3:** Multivariate analysis of the probability of survival over 15 months over variables age, type of ALS, PEG implantation and nutritional status measured by SGA.

SURVIVAL > 12 Months	Odds Ratio	CI 95%	*p*-Value
PEG (Yes/No)	6.81	2.05–22.68	<0.01
Age (years)	1.01	0.966–1.06	0.65
Type of ALS (Bulbar/Spinal)	0.47	0.16–1.13	0.15
Nutritional Status (Malnourished (SGA:B-C)/Well nourished (SGA:A)	0.47	0.16–1.37	0.17

CI, confidence interval; ALS, amyotrophic lateral sclerosis; PEG, percutaneous endoscopic gastrostomy; SGA, subjective global assessment.
